# Peripheral blood T cells and neutrophils from asthma patients express class-I MHC-restricted T cell-associated molecule

**DOI:** 10.1186/1710-1492-10-46

**Published:** 2014-09-02

**Authors:** Carlos Ramirez-Velazquez, Nonantzin Beristain-Covarrubias, Leopoldo Guido-Bayardo, Vianney Ortiz-Navarrete

**Affiliations:** Molecular Biomedicine Department, Centro de Investigación y de Estudios Avanzados (CINVESTAV)-IPN, Av. IPN No. 2508, Colonia San Pedro Zacatenco, México; Allergy Department, Hospital General Dr. Fernando Quiroz Gutiérrez, ISSSTE. Calle Felipe Angeles y Canario. Colonia Bellavista, Mexico, DF CP 01140 Mexico; Allergy Department, Centro Médico Nacional 20 de Noviembre ISSSTE, Felix Cuevas 540, Colonia del Valle, Mexico, DF CP 03229 Mexico

**Keywords:** CRTAM, CD355, Asthma, CD4 T cell, CD8 T cells, Neutrophils, Eosinophils

## Abstract

**Background:**

Class-I MHC-restricted T cell-associated molecule (CRTAM) is a protein expressed by activated natural killer T (NKT) cells, natural killer (NK) cells, CD8 T cells, and certain CD4 T lymphocytes. It is also expressed in Purkinje neurons and epithelial cells. However, no studies have examined the expression of CRTAM in peripheral blood cells during homeostasis or disease. Therefore, we explored whether CRTAM expression is influenced by the presence of allergic asthma.

**Methods:**

We collected whole peripheral blood cells from non-asthmatic control subjects (n = 17) and patients with asthma (n = 17). All patients with asthma tested positive in allergen skin prick tests. We analyzed CRTAM expression in CD4^+^ and CD8^+^ T lymphocyte populations. CRTAM expression was also analyzed in CD177^+^ neutrophils and IL5Rα^+^ eosinophils.

**Findings:**

The percentage of CD4^+^CRTAM^+^ and CD8^+^CRTAM^+^T lymphocytes in peripheral blood was higher in allergic asthma patients compared with healthy controls. Furthermore, the percentage of CD177^+^CRTAM^+^ neutrophils in peripheral blood was also elevated in patients with allergic asthma. However, the percentage of IL5Rα^+^CRTAM^+^ eosinophils in peripheral blood was not significantly different in patients with allergic asthma compared with healthy controls.

**Conclusions:**

CRTAM expression on T cells, eosinophils, and neutrophils may be involved in bronchial inflammation in allergic asthma. Determination of CRTAM expression in peripheral blood may be useful for the diagnosis of bronchial inflammation and/or to identify recently activated immune cells.

## Background

Asthma is a problem worldwide, with an estimated 300 million affected individuals [1]. It is a heterogeneous chronic inflammatory respiratory disease that is characterized by mucus overproduction and airway-wall remodeling that results in bronchial hyperactivity and airway obstruction [2]. Allergens and some pathogens have been implicated in the worsening of asthma. The characteristic patterns of inflammation found in allergic diseases is observed in asthma, including activated mast cells, increased numbers of activated eosinophils, increased numbers of invariant natural killer T cells (NKT), T helper 2 and 17 lymphocytes (Th2 and Th17), and neutrophils. Each of these cell populations releases mediators, contributing to symptoms or enhancing resistance to steroid treatment. Structural cells of the airways also produce inflammatory mediators and contribute to the persistence of inflammation in various ways. Over 100 deterrent mediators are now recognized to be involved in asthma and mediate the complex airway inflammatory response [3–6].

Asthma diagnosis and management is generally based on reported asthma symptoms and is often combined with lung function tests to assess reversible airway obstruction and airway hyperresponsiveness [2]. However, symptoms and lung function measurements may not reflect the underlying airway inflammation. Bronchoscopy with biopsies and bronchoalveolar lavage (BAL) are considered the best methods to assess airway inflammation. However, they are too invasive for general application in clinical practice [7]. Although the clinical value of a single FeNO measurement is limited, combining this measure with other markers of airway inflammation may lead to more accurate assessments of disease stage [8]. In addition, asthma appears to encompass a broad collection of heterogeneous disease subtypes with different underlying pathophysiological mechanisms [9]. Thus, there is a need for asthma biomarkers in order to identify relevant clinical asthma phenotypes, optimize diagnosis, and guide treatment.

Class-I MHC-restricted T cell-associated molecule (CRTAM) was initially described as a protein expressed only on activated NKT cells and CD8 T cells [10, 11]. This protein, named CD355 during The 9th HLAD workshops [12], has also been identified in a small fraction of phorbol 12-myristate 13-acetate (PMA)/ionomycin-activated CD4 T cells. The interaction of CRTAM with its ligand, Necl-2, promotes cell adhesion, NK cell cytotoxicity, and IFNγ-secretion by CD8 T cells [13, 14]. Non-lymphoid cells also express CRTAM, such as Purkinje neurons or epithelial cells, where it is involved in epithelial cell adhesion [11, 15]. CRTAM is also essential for the establishment CD4 T cell polarization after TCR engagement and induce the capacity to secrete IFNγ, IL-17 and IL-22 [16]. However, no studies have investigated CRTAM expression in peripheral blood cells in a normal state or during disease (e.g., allergic asthma). Taken in consideration that recently activated immune cells express CRTAM it would be valuable to study its expression in patients with asthma. In this study, we analyzed whether CRTAM is expressed in human neutrophils, eosinophils, CD4 T cells, and CD8 T cells from allergic asthmatic patients.

## Methods

### Patients and control subjects

We recruited seventeen patients with asthma diagnosis according to Global Strategy for Asthma Management and Prevention: GINA Executive Summary 2008 [17] (Table 1). All patients tested positive to at least one allergen (house dust mites, pollens, or fungal allergens) in allergen skin prick tests (>5 mm; Alerquim, Mexico City, Mexico). Among them we found seven patients with mild asthma, four patients with moderate but persistent asthma and six patients with acute asthma; classified according to GINA. The acute asthma patients were defined as those who show exacerbation in symptoms such as wheezing, breathlessness, and chest tightness 48 hours prior to admission to the emergency department and received only rescue medication. These patients were enrolled within 24 hours of admission to the emergency department. Prior to the start of treatment, a blood sample was obtained for this study. Nine asthmatic patients had treatment with allergen- specific immunotherapy for more than 6 months (wt/vol;Alerquim, Mexico City). All subjects were either nonsmokers or ex-smokers, who had quit smoking for at least 12 months prior to the study. Subjects who had used corticosteroids, long-acting β2-agonists, leukotriene antagonists, or antihistamines in the month preceding the study were excluded, along with subjects with history of respiratory tract infection within the 4 weeks preceding the study. Healthy subjects without history of allergy or bronchial symptoms and who tested negative in allergen skin prick tests (Alerquim) comprised the control group. We measured total serum immunoglobulin E and the forced expiratory volume in 1 second (FEV1) in every subject. Three different independent measurements of FEV1 were performed with a dry spirometer (Medgraphics, Minnesota, USA). The optimum value is expressed as a percentage of the predicted value. All asthmatic patients had 12% of reversibility in FEV1 after 200-μg salbutamol [17]. The Ethics Committee of the Fernando Quiroz Hospital approved the study, and each subject gave written informed consent.

**Table 1:** **Characteristics of study subjects**

	Asthma patients	Healthy controls
Sex (female/male)	6/11	7/10
Age, y (mean ± SEM)	22.35 ± 3.82	24.12 ± 1.38
Atopy (N°)^1^	17/17	0/17
Total serum IgE levels (IU/mL) (mean ± SEM)	425.2 ± 105.2***	278.89 ± 14.6
FEV1 (% predicted; mean ± SEM)	77.65% ± 4.77***	96.59% ± 1.61

^1^Atopy is defined as at least one positive prick test.

***P < 0.001 compared with non-asthmatic controls.

FEV1: forced expiratory volume in 1 second; Ig, immunoglobulin; SEM, standard error of the mean.

### Preparation of human mononuclear cells

Whole blood cells were obtained from 17 healthy volunteers and 17 patients with asthma. Peripheral blood mononuclear cells (PBMCs) were isolated using a differential centrifugation gradient (Ficoll-Paque PLUS, GE Healthcare). PBMCs were analyzed for viability using trypan blue, washed, and stained *ex vivo*.

### Surface staining

*Ex vivo* cells from heparinized whole blood (HWB) were stained for 20 min at 4°C with fluorescein isothiocyanate (FITC)-conjugated anti-CD177, phycoerythrin (PE)-conjugated anti-IL-5Rα, and allophycocyanin (APC)-conjugated anti-CRTAM (R&D Systems). Blood erythrocytes were lysed for 15 min at room temperature (RT) in lysis buffer solution [155 mM NH_4_C1, 10 mM KHCO_3_, and 0.1 mM EDTA, (pH 7.3)], and analyzed using a CyAn ADP cytometer (Beckman Coulter, Inc. Indianapolis). PBMCs were stained for 20 min at 4°C with FITC-conjugated anti-CD3, allophycocyanin (APC)-Cy7-conjugated anti-CD4, PE-conjugated anti-CD8 (BioLegend), and (APC)-conjugated anti-CRTAM (R&D Systems) and subsequently analyzed using a CyAn ADP cytometer. Isotype control matched monoclonal antibodies were used as negative controls for each fluorochrome.

### Flow cytometry analyses

Neutrophils were identified according to size (forward scatter, FSC) and complexity (side scatter, SSC), and the expression of CD177 (BioLegend). The eosinophil IL5Rα marker was used to distinguish eosinophils from neutrophils in HWB to further evaluate CRTAM expression in neutrophils and eosinophils. CRTAM expression was also evaluated in CD3^+^CD4^+^ and CD3^+^CD8^+^ lymphocytes from PBMCs previously gated according to FSC and SSC. Data analyses were performed using FlowJo 7.6.5 software.

### Statistical analyses

Distributions of continuous variables are expressed as the mean ± standard error of the mean (SEM) and median. Nonparametric Mann–Whitney U tests were used to compare continuous variables, Wilcoxon tests were used for comparisons among two groups. Friedman’s post hoc tests were used to confirm differences in individual groups. P values less than 0.05 were considered statistically significant.

## Findings

### CRTAM expression in peripheral blood T lymphocytes

**Figure 1:**
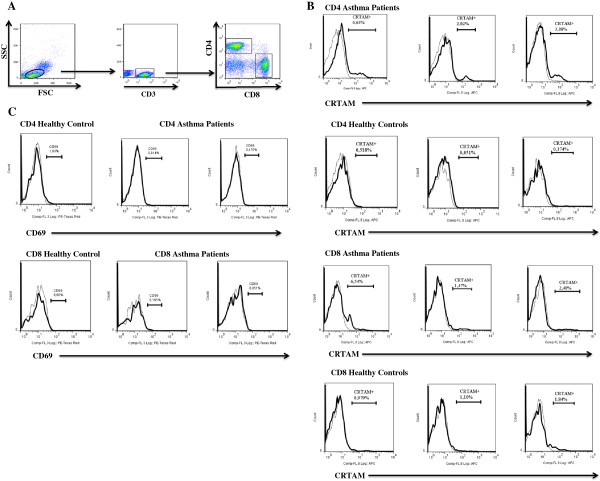
**Peripheral blood CD4 T cells and CD8 T cells from asthma patients express CRTAM.** **(A)** A representative dot plot is shown from one patient with asthma. **(B)** CRTAM expression was analyzed in CD4+ and CD8+ populations from patients with asthma and healthy controls. A representative histogram is show from three asthmatic patients and three healthy controls. Dashed lines show the staining of isotype control monoclonal antibodies. **(C)** CD69 expression was analyzed in CD4+ and CD8+ populations from patients with asthma and healthy controls. A representative histogram is shown from two asthmatic patients and one healthy control. Dashed lines show the staining of isotype control monoclonal antibodies.

**Figure 2:**
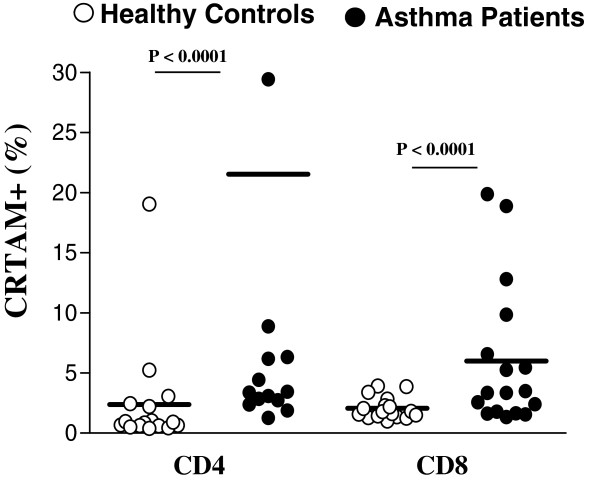
**CRTAM** ^**+**^ **CD4 T cells and CRTAM** ^**+**^ **CD8 T cell frequency in peripheral blood from asthma patients and healthy controls.** CRTAM expression was determined by flow cytometry as shown in Figure 1B.

PBMCs were isolated from peripheral blood of allergic asthma patients and healthy controls. Cells were analyzed by flow cytometry according to size (forward scatter, FSC) and complexity (side scatter, SSC), as well as by gating CD3^+^, CD4^+^, and CD8^+^ populations (Figure 1A). Analyses were performed using 4-color flow cytometry based on CRTAM expression in CD4^+^ and CD8^+^ T lymphocyte populations (Figure 1B). We observed that the percentage of CD3^+^CD4^+^CRTAM^+^ (21.53% ± 7.4% vs. 2.37% ± 1.08%, P < 0.0001) and CD3^+^CD8^+^CRTAM^+^ (5.98% ± 1.43% vs. 2.06% ± 0.22%, P < 0.0001) T lymphocytes was higher in allergic asthma patients compared with healthy controls subjects (Figure 2). Remarkable we observed a very low percentage CD4+ and CD8+ T cells that expressed the T cell activation marker CD69 for both asthma patients and healthy controls (Figure 1C). CD3 + CD4+ CD69+ (0.6857% ± 0.1060% vs 0.8803% ± 0.1394%,) and CD3 + CD8+ CD69+ (0.07112% ± 0.03457% vs 0.06594% ± 0.01767%,) and it was not difference (P < 0.72)

### CRTAM expression in peripheral blood neutrophils and eosinophils

**Figure 3:**
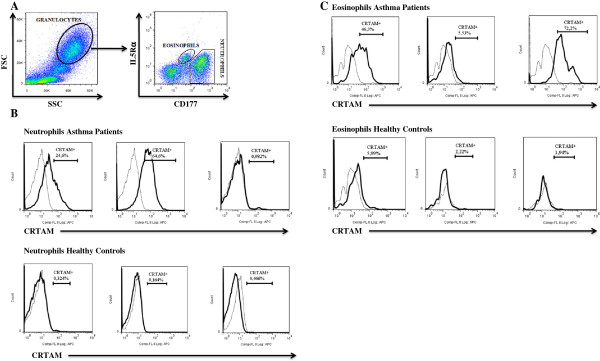
**Peripheral blood neutrophils and eosinophils from asthma patients express CRTAM. (A)** A representative dot plot is shown from a patient with asthma. **(B)** CRTAM expression was analyzed in neutrophils from patients with asthma and healthy controls. **(C)** CRTAM expression was analyzed in eosinophils from patients with asthma and healthy controls. A representative histogram is shown from three patients with asthma and three healthy controls. *Dashed lines* show the staining of isotype control monoclonal antibodies.

**Figure 4:**
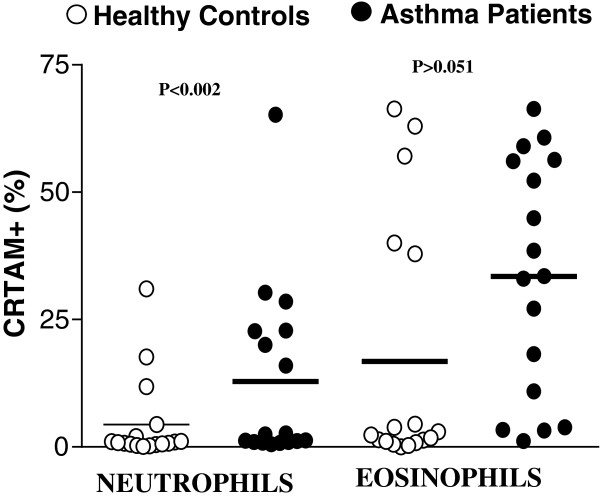
**CRTAM** ^**+**^ **neutrophil and CRTAM** ^**+**^ **eosinophil frequency in peripheral blood from asthma patients and healthy controls.** CRTAM expression was determined by flow cytometry as described in Figure 3B.

We analyzed neutrophils and eosinophils in HWB from allergic asthma patients and healthy controls by flow cytometry according to size (forward scatter, FSC) and complexity (side scatter, SSC), as well as the expression of CD177 and IL5Rα (Figure 3A). CRTAM expression was evaluated in the CD177^+^ (Figure 3B) and IL5Rα^+^ (Figure 3C) population. We observed that the percentage of CRTAM + neutrophils (12.84% ± 4.23% vs. 4.38% ± 2.02%, P < 0.002) was significantly increased in patients with allergic asthma compared with healthy control subjects. However, the percentage of CRTAM + eosinophils (33.45% ± 5.6% vs. 16.78% ± 6.04%, P > 0.051) did not differ in patients with allergic asthma compared with healthy controls (Figure 4).

## Discussion

Asthma biomarkers in peripheral blood are easy to obtain, and the procedure, itself, is less invasive than sputum induction and bronchoalveolar lavage (BAL). Because inflamed tissues release chemoattractants and cytokines that recruit activated immune cells from the peripheral blood, the dynamic process of immune cells entering and leaving the bloodstream can be used as an indirect readout of disease state [7].

Many studies have shown that inflammatory cells, such as monocytes and granulocytes, respond to inflammatory signals by upregulating several activation markers [6, 18, 19]. Many of these markers, including CD11b/CD18 (Mac-1), CD63, CD66, and CD67, are typically found in granules that fuse with the plasma membrane upon activation of the cells with inflammatory mediators [20]. However, previous studies that compared the presence of markers on blood cells and tissue cells obtained from sputum and BAL did not take into account that cells homing to the tissue under homeostatic conditions exhibit the same phenotype [21, 22]. Thus, the expression of these markers in peripheral blood has not led to a clear link between granulocyte expression profiles and asthma type.

In this study, we found an increase in peripheral blood neutrophils that express cell surface CRTAM from patients with allergic asthma. Peripheral blood eosinophils expressing CRTAM were also increased in patients with allergic asthma but the difference was not significant (P = 0.051). No differences were noted between the severity of disease (acute, mild or moderate asthma) and CRTAM expression or treatment with specific immunotherapy (data not shown). It is currently not known what induces CRTAM expression in these polymorphonuclear leukocytes or how CRTAM functions; however, because it belongs to a family of proteins involved in cell adhesion, it is likely that CRTAM is involved in leukocyte transmigration through venular walls. Further studies will be necessary to define these fundamental features. We also observed an increase in CRTAM expression in CD4 T cells and CD8 T cells in peripheral blood from allergic asthma patients but it was not associated with the clinical features evaluated (severity or a specific immunotherapy). It has been reported that CRTAM expression is driven by TCR/JNK pathway [23]. Therefore, it is likely that allergens presented by MHC molecules stimulate surface expression of CRTAM on T cells from patients with asthma and the interaction of CRTAM with its ligand Nelc-2 might participate in Th1 and/or Th2 polarization, as has been described for mouse T cells [16]. However the frequency observed of CD3 + CD4+ CRTAM + T cells represent more than 20%. It is very likely that among this CRTAM + subpopulation are included antigen specific and also a bystander activated CD4 T cells. Future studies are need to determine whether expression of CRTAM is induced by another stimulus (e.g. cytokines or chemokines). In this context recently have been published that CRTAM gene associate with an increase of asthma exacerbation and the presence of a low circulating vitamin D levels. Studies on cell lines confirmed the influence of vitamin D and CRTAM gene expression [24]. Suggesting that others signaling pathways play a role for the expression of CRTAM. Further studies are required to elucidate these important aspects on CRTAM expression and whether it might contribute during the initial phase of asthma disease and/or during progression.

## Conclusions

In conclusion, we propose that CRTAM expression on T cells, eosinophils, and neutrophils may be involved in bronchial inflammation in allergic asthma. Expression of CRTAM in peripheral blood may be useful for the diagnosis of bronchial inflammation or to identify recently activated immune cells.

## Abbreviations

PMA: Phorbol myristate acetate
FACS: Flow cytometry (fluorescenceactivated cell sorting)
SEM: Standard error of the mean
FSC: Forward scatter
SSC: Side scatter
PBMC: Peripheral blood mononuclear cell
HLDA9: Ninth International Workshop on Human Leukocyte Differentiation Antigens.
